# A Review of the Mechanism of Action of Drugs Used in Congestive Heart Failure in Pediatrics

**DOI:** 10.7759/cureus.33811

**Published:** 2023-01-16

**Authors:** Rakshit K Singh, Revat J Meshram, Aakriti Tiwari

**Affiliations:** 1 Department of Pediatrics, Jawaharlal Nehru Medical College, Datta Meghe Institute of Higher Education and Research, Wardha, IND

**Keywords:** aldosterone antagonists, ace inhibitors and angiotensin receptor blockers, ishlt guidelines, sglt-2 inhibitor, digoxin, heart failure

## Abstract

Congestive heart failure (CHF) is a complex, heterogeneous medically ill condition that can occur due to diverse primary (cardiomyopathies, coronary artery diseases, and hypertension) and secondary causes (high salt intake and noncompliance toward treatment) and leads to significant morbidity and mortality. The approach toward managing the patient of CHF in the pediatric age group is more complex than in the adult population. Currently, in the adult group of the population of CHF, there are well-established guidelines for managing these patients, but in the case of children, there are no well-established guidelines; therefore, this systematic review gives more ideas for managing the pediatric population undergoing CHF. Treatment of the underlying cause, rectification of any advancing event, and management of pulmonary or systemic obstruction are the principles for management. The most widely used drugs are diuretics and angiotensin-converting enzyme (ACE) inhibitors, whereas beta-blockers are less commonly used in children than in adults. ACE inhibitors such as captopril, enalapril, and cilazapril are widely used in the pediatric age group. ACE inhibitors act on the renin-angiotensin-aldosterone system (RAAS) similar to those in the adult population. In children with heart failure (HF), ACE inhibitors reduce the pressure in the aorta, resistance in the systemic blood vessels, and upper left and right chamber pressures but do not appreciably influence pulmonary vascular resistance. We use a patient's initial perfusion and volume status assessment to decide further action for the supervision of acute HF. This paradigm was adopted from adult studies that showed higher rates of morbidity and mortality in patients with HF whose hemodynamic or volume status assessment results were stable with a pulmonary capillary wedge pressure >18 mmHg and a combined index (CI) of 2.2 L/minute/m^2^. ACE inhibitors, beta-blockers, and spironolactone are the most widely prescribed drugs for the chronic condition of CHF. This study shows the current status of medical therapy for critical as well as persistent pediatric HF.

## Introduction and background

Heart failure (HF) is a complex clinical condition that results from any structural and functional cardiac disease that leads to an inability to fill the heart chamber or propel the blood forward [1]. Congenital heart disease (CHD) is the most important reason for congestive HF (CHF) in children [1]. Depending on the etiology, drug therapy is used to either shorten the time awaiting necessary curative surgery or manage HF on a long-term basis. In people with CHF, beta-blockers are lifesaving; therefore, they are a staple of routine care. The major effect and adverse outcomes of HF may differ depending on the underlying causes, which may differ from causes in the case of adults. Additionally, the dosage may need to be carefully modified for various age groups [2]. HF is a disorder that has a high morbidity and mortality rate, although having an estimated incidence of 0.9-7.4 per 100,000 children [3,4]. Sixty-four percent of HF admission in patients under the age of 18 years in the modern period occurs in newborns [3]. CHF is not a disease condition but rather a pathophysiological state in which an aberration of heart function is answerable for the malfunction of the heart to propel blood at a pace proportionate with the necessities of the processing tissues. The main causes of CHF are ischemic heart disease (IHD), rheumatic heart disease (RHD), and advancing age. There are additional subtle indicators as well, some of which are signs of declining cardiac function and others of which are etiologic [4]. Fetal cardiomyopathies or extracardiac disorders are the primary causes of HF at birth (such as infection, low glycemic index, and low levels of calcium). The main cause of CHD in the first week of birth is ductus-dependent circulation, which occurs in conditions such as severe congestion in the coarctation of the aorta and hypoplastic left heart disorder when the ductus arteriosus closes and severely reduces end-organ perfusion. In the first month of life, congestive heart disease with the left to right shunts, such as ventricular septal defect (VSD), patent ductus arteriosus (PDA), and aortopulmonary windows, are responsible for pediatric HF. In these conditions, pulmonary blood flow increases gradually as pulmonary resistance decreases. Last but not the least, HF in adolescents is more frequently associated with cardiomyopathies or myocarditis rather than secondary to CHD [5].

Depending on the systolic function, there are two main types of HF:

· HF with reduced ejection fraction (HFrEF) or left ventricular ejection fraction (LVEF) <40%

· HF with preserved ejection fraction (HFpEF) or LVEF >= 50%

HF results in a decrease in cardiomyocyte contractility, regardless of the etiology. A reduction in cardiac output caused by the original injury is then compensated by two main compensatory mechanisms. The first mechanism is the activation of the sympathetic nervous system, which causes a rise in norepinephrine discharge and diminished norepinephrine uptake as well as peripheral vasoconstriction to preserve the mean pressure of arteries and perfusion of organs (by raising systemic vascular resistance). However, elevated catecholamine levels trigger further cardiomyocyte damage, abnormal signaling in the cell, and eventually cardiomyocyte death. The activation of the renin-angiotensin-aldosterone system (RAAS), which involves elevated levels of renin, angiotensin II, and aldosterone in the blood is the second significant compensatory mechanism. Angiotensinogen is broken down by renin into angiotensin I, which is then transformed by the angiotensin-converting enzyme (ACE) into angiotensin II. A powerful vasoconstrictor angiotensin II maintains end-organ perfusion. The effects of aldosterone on salt and water maintenance include amplified preload and cardiac output [5]. Modified Ross Classification for pediatric HF is given in Table 1.

**Table 1:** Modified Ross classification for pediatric heart failure. Author’s own creation. Adapted from [5].

Classes	Symptoms
Class 1	Asymptomatic
Class 2	Mild tachycardia
Class 3	Marked tachypnoea
Class 4	Symptoms like tachypnoea, grunting, or diaphoresis at rest

Dyspnea (shortness of breath), fatigue, and common clinical manifestations such as pulmonary rales, peripheral edema, or distended jugular veins are all indicators of HF [6]. The 2014 Prospective Comparison of Angiotensin Receptor/Neprilysin Inhibitor (ARNI) with Angiotensin II Type 1 Receptor Blocker (ACEI) to Determine Impact on Global Mortality and Morbidity in HF (PARADIGM-HF) trial found that ARNI and valsartan were more effective than ACE inhibitor enalapril in reducing morbidity and mortality in patients with chronic HFrEF [7]. There is some debate regarding the precise mechanisms causing the therapeutic advantage of neprilysin inhibitors. It is unknown which of the several neprilysin substrates such as physiologically active natriuretic peptides, adrenomedullin, endothelin, angiotensin II, or a mix of substrates is responsible for the reported benefits. The pathophysiologic heterogeneity of the illness is a significant barrier impeding the discovery of new treatments for HF. Many pathophysiologic changes can lead to the clinical state of HF, such as ischemia, infarction, volume overload, dysregulation of metabolism, gene mutations that affect the way the protein of sarcomeres functions, and reaction to viral infections. Several prime causes of HF are linked to both confined and complete activation of inflammatory signaling pathways [8].

Given that, almost all medications that have been shown to improve prognosis have an impact on these factors as well as renal function, blood pressure, and ionic balance. It is intriguing to think about how the delivery of this treatment might be optimized in light of the unique characteristics of each patient. Furthermore, given that there is no convincing cause to begin lifesaving medications in a step-by-step manner, it is reasonable to begin medications with a demonstrated prognostic benefit simultaneously to administer the various agents by their pharmacodynamic effects and patient characteristics. With this strategy, HF medications could be escalated more quickly to provide the patient with the greatest possible benefits [9]. Clinical manifestations, congestion levels, hemodynamic state, and renal function vary across HF patients. This will enable the tailored application of lifesaving treatment by altering or prioritizing therapies by the hemodynamic and renal phenotypic profile. The majority of patients who come with HF frequently have preexisting problems and were previously using RAAS inhibitors and beta-blockers, although a progressive approach to the adoption of HF medicines can be excused in drug-naive individuals. The difficult part is deciding which drugs to up titrate and in what order. When implementing HF therapy, heart rate, blood pressure, kidney function, and their groupings are the most crucial factors to take into account [9].

## Review

Methodology

We undertook a systematic search through PubMed Central in November 2020 using keywords such as HF, beta-blockers, diuretics, ACE inhibitors, aldosterone antagonists, G-protein-coupled receptor antagonists, digoxin, wet and cold patients, catecholamines, phosphodiesterase inhibitors, calcium sensitizers, action cardiac therapy improving outcome network (ACTION). We additionally searched for key references from bibliographies of the relevant studies. The search was updated in February 2022. One reviewer independently monitored the retrieved studies against inclusion criteria, in the beginning, based on the title and abstract and then on full texts. Another reviewer also reviewed approximately 20% of these studies to validate the inclusion of studies.

Mechanism of action

The purpose of clinical therapy for CHF in a child is to promote recovery, preserve steadiness, limit disease progression, and create a conducive environment for somatic growth and the best possible development of the child into an adolescent. ACE inhibitors, beta-blockers, diuretics, and aldosterone antagonists are typically used in combination [10].

ACE Inhibitors and Aldosterone Antagonists

**Figure 1:**
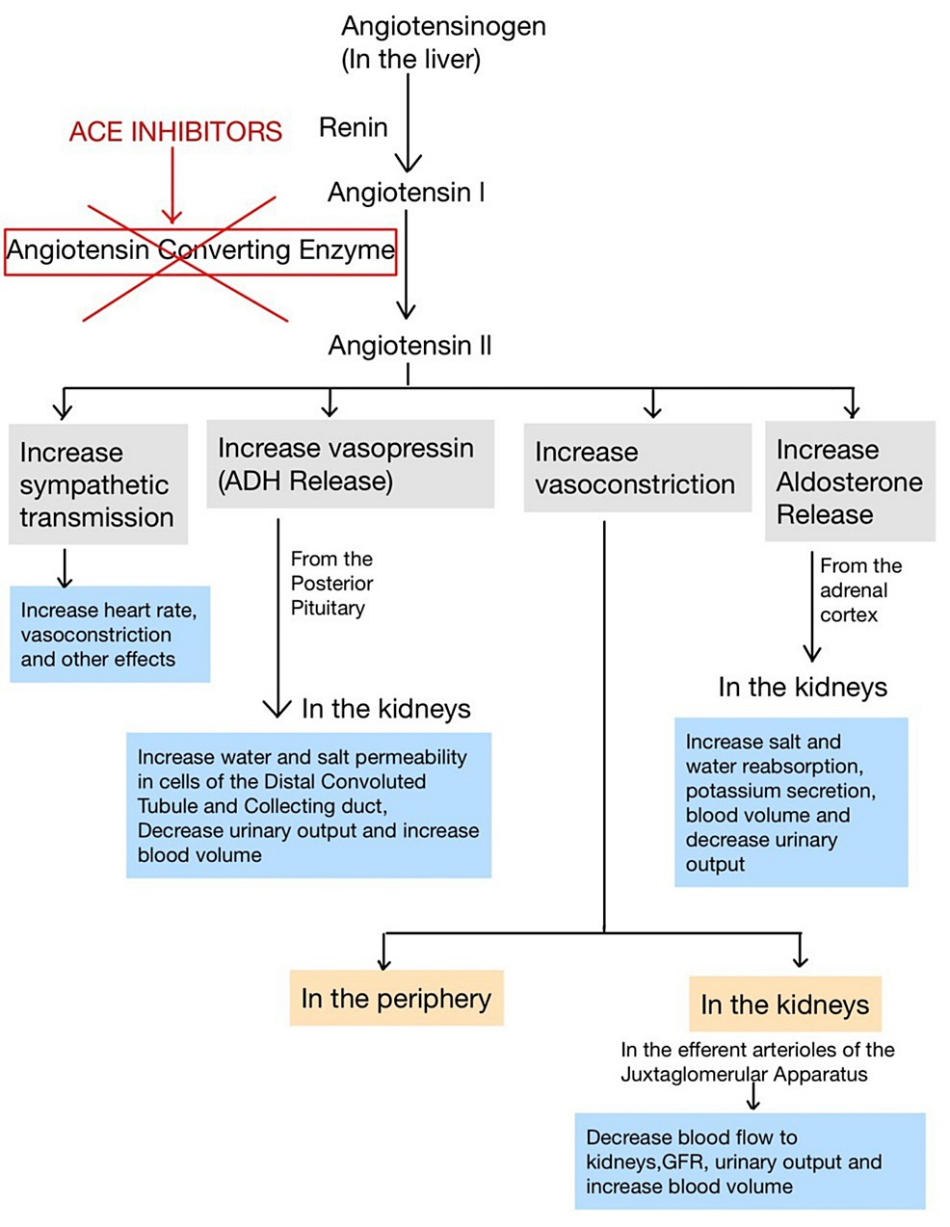
Mechanism of action of ACE inhibitors. Illustrated by authors. Source: [11] ACE, angiotensin-converting enzyme; ADH, antidiuretic hormone; GFR, glomerular filtration

CHF disease with diminished systolic function is characterized by activation of the RAAS pathway, which is a physiological response to reduce renal perfusion. Renin promotes the process by which hepatic angiotensinogen is transformed into angiotensin I, which is then processed by the ACE, primarily in the lungs, to create angiotensin II, a powerful constrictor of the blood vessels that has long-term detrimental consequences on HF. The mechanism of action of ACE inhibitors is shown in Figure 1. This can be due to the release of aldosterone, which increases sodium reabsorption, and vasoconstriction of renal afferent arterioles. Vasoconstriction induces the hypertrophy of cardiomyocytes and death when the RAAS activation is not inhibited [11]. ACE inhibitors can reverse remodeling, reduce vascular resistance, and enhance vascular compliance. Particularly with ACE inhibitors that have significant tissue penetration, like lisinopril, the remodeling properties appear to be higher [12]. Their beneficial hemodynamic changes comprise a decrease in cardiac preload, afterload, and systolic ventricular wall stress, thereby leading to increased cardiac output without a corresponding rise in oxygen consumption [13]. This hemodynamic change enhances renal perfusion and encourages salt excretion, thereby maintaining glomerular filtration. As a result, they play a crucial role in the long-term care of patients with CHF. Bradykinin breakdown can be stopped with ACE inhibitors, which may increase kinin-induced peripheral vasodilation and enhance organ perfusion. However, these medications can infrequently cause moderate renal insufficiency as a result of a decrease in the glomerular filtration rate (GFR). This functional renal insufficiency frequently arises when the GFR is extremely angiotensin-II-dependent, such as in situations like low-volume, bilateral renal artery stenosis, in a single functional kidney, as in transplant recipients. It can also occur when renal perfusion is reduced as a result of a decline in mean arterial pressure [13].

The synthetic steroid spironolactone, a commonly used mineralocorticoid antagonist in pediatrics, reduces aldosterone's capacity to bind to its receptor in the distal renal tubule in a competitive manner, causing sodium to be excreted while potassium is preserved. Spironolactone has a very modest diuretic impact, but because of its potassium-saving properties, it is very effective in treating acute HF patients [3].

Beta-Blockers

Beta-blockers are the mainstay treatment in the management of CHF with reduced ejection fraction. Different drugs have varying selectiveness for beta 1 or 2 receptors: some only partially activate the receptors (inherent sympathomimetic action), some have supplementary belongings on adrenoceptors, or they may stimulate the production of nitric oxide [14]. The majority of commonly used beta-blockers (metoprolol, carvedilol, propranolol, nebivolol, and bisoprolol) are contrary agonists at the beta 1 adrenoceptor, which means that exposure to the drug reduces a predominating basal level and constitutionally decreased signal transduction from the receptor even in the absence of an agonist for the receptor [15].

Diuretics

The prototypical diuretics are furosemide, bumetanide, and torsemide bind to the outside of the cell surface of the sodium-potassium chloride symporter, directly inhibiting ion transport [16]. These drugs inhibit the symporter at the thick ascending part of the loop of Henle, inhibiting sodium-potassium chloride cotransporter 1 (NKCC1), which is present on gene *SLC12A2* (solute carrier family 12 members 2), a second sodium-potassium chloride symporter isoform that is highly expressed in every part of the body and also in the ear, is another feature of loop diuretics that contributes to its ototoxicity [17]. Loop diuretics cause dilatation of blood vessels when intravenously delivered, by preventing NKCC1 in vascular smooth muscle cells [18]. Cells of the extraglomerular mesangium and afferent arteriole in the kidney both express NKCC1, which inhibits basal renin secretion [19]. In contrast to other treatments for HF, loop diuretics aim to induce a short-term negative salt water balance (decongestion), as well as a long-term reduction in extracellular fluid volume. Loop diuretics' effects on the extracellular fluid volume are complicated because their half-lives are shorter than those of common dosing intervals (typically twice daily) and because they principally impede solute transport via just one of multiple sodium reabsorbing nephron segments [20].

Sodium-Glucose Cotransporter 2 Inhibitors

Empagliflozin, dapagliflozin, and canagliflozin are widely available SGLT2 Inhibitors that act by their osmotic diuresis and natriuretic effect, contributing to the decrease in plasma volume and drop in systolic pressure and diastolic pressure by 4 to 6 and 1 to 2 mmHg, respectively [21]. Although the SGLT2 reserve has shown promise as a new protective therapy for the heart, it is crucial to be aware of any potential side effects associated with medications in this class, such as elevated rates of sepsis, postural hypotension, polyuria, diabetic ketoacidosis, acute kidney injury, and perhaps elevated chances of bone cracks. Genital fungal infection is one of the most frequent side effects of SGLT2 inhibitors. This can be diminished by practicing adequate genital hygiene. Due to the increased risk of perineal necrotizing fasciitis, people who are immobile or incontinent, have intertrigo in the groin area, have chronic diarrhea, or are incapable of maintaining good genital hygiene should refrain from using SGLT2 inhibitors [22]. In individuals with inadequate insulin, low sugar intake, volume depletion, heavy alcohol intake, or concurrent illnesses, euglycemic diabetic ketoacidosis, an uncommon but severe contrary effect of SGLT2 inhibitors, might develop [23].

G-Protein-Coupled Receptor

The most common class of surface receptors that are the target of many medications is the G-protein-coupled receptor (GPCR), which plays a crucial role in the control of heart functioning in both healthy and sick people. Adrenergic and angiotensin II receptors are examples of the many GPCR antagonists that are now accepted as standard therapy for a variety of disorders, such as hypertension, coronary artery disease, and cardiac failure. Guanine nucleotide-binding proteins (G proteins) allow GPCR to transmit signals. G proteins are so termed because they can bind the nucleosides guanosine triphosphate (GTP) and guanosine diphosphate (GDP). When G proteins are attached to GTP, they are activated, and when bound to GDP, they get inactive. They serve as a molecular switch in the transmission of signaling into the cell [24]. Currently, there are 21 G alpha subunits, 6 G beta subunits, and 12 G gamma subunits. G alpha subunit is the type of membrane-associated, heterotrimeric G protein, which is further split into four primary classes: G stimulatory (Gs), G inhibitory (Gi), G alpha q, and G alpha 12/13 [25].

The second messenger, cyclic adenosine monophosphate (cAMP), is produced by the effector enzyme adenylyl cyclase in response to the G stimulatory. PKA is then stimulated, and a variety of intracellular proteins regulate cellular response [26]. Gi, on the other side, prevents adenylyl cyclase, hence reducing intracellular cAMP [27]. The second messenger inositol 1,4,5-triphosphate (IP3) and diacylglycerol are produced when Gq activates the enzyme phospholipase C, which, in turn, breaks down the membrane-bound second messenger phosphatidylinositol 4,5-bisphosphate. In the endoplasmic reticulum, inositol triphosphate encourages calcium release. Protein kinase C (PKC) will be activated by elevating intracellular calcium that diffuses from the plasma membrane, promoting cellular signaling [27,28]. Different from cell surface receptors, GPCR found in other cellular compartments can also trigger subsequent signaling. In early endosomes, for instance, the beta 2 adrenergic receptor (β2AR) stimulates Gs to encourage cAMP synthesis [29]. Adenylyl cyclase is activated by the beta 1 adrenergic receptor (β1AR) on the nuclear membrane of the cardiomyocyte [30]. Endothelin stimulates the endothelin receptors that are positioned in the nucleus to change the amount of nuclear calcium [31] and the nuclear β1AR activates extracellular signal-regulated kinase (ERK), which is located in the plasma membrane caveolae [32].

Digoxin

Digoxin, a glycoside for the heart, served as the mainstay of HF management for many years before a hypothesis shift in HF pathophysiology resulted in a switch from inotropic therapy to neurohormonal control. Digoxin prevents sodium from being removed from the myocytes during the potassium exchange by binding to the sarcolemma sodium-potassium ATPase pump. In turn, the sodium-calcium exchanger can accept more sodium intracellularly, allowing for an influx of calcium. A more powerful mechanical contraction is aided by the transport of calcium into the sarcoplasmic reticulum. Additionally, some studies indicate that digoxin binds to ryanodine receptor 2 [33]. The Digitalis Investigation Group randomized, double-blind, placebo-measured experiment, which included 5,800 patients, concluded that adding digoxin to ACE inhibitors and diuretics had no outcome on overall mortality [34]. The implantation of an umbilical artery catheter (UAC) is the most frequent renovascular defect linked to hypertension in neonates [35]. Although genuine thrombi were not identified in renal arteries, hypertension seemed to develop in newborns with UAC. As a result, it is believed that catheter-associated hypertension is related to the formation of thrombus related to blood vessels, to which preterm newborns may be extremely susceptible. A longer duration of placement of the catheter leads to increased chances of the formation of a thrombus [36]. With an HF phenotype that is cell-typical, persistent tachycardia causes systolic HF. Within days to weeks, there will be an increase in wall tension, a rise in wall dilatation, reduction in systolic contractility, and decline in cardiac output [37].

Wet and cold patients 

Wet Patients

Under the most recent ISHLT guidelines, there is a Class I indication for the initiation of diuretics in patients presenting with fluid retention coupled with ventricular dysfunction for the management of pediatric HF. The use of diuretics should continue until euvolemia is reached.

Cold Patients

When pediatric patients present in cardiogenic shock, there is a Class I indication for temporary use of intravenous inotropic support [3].

Drugs for acute HF

Catecholamines

Adrenergic receptors play a dose-dependent and mediating role in catecholamines' hemodynamic effects. Adrenergic receptors are closely linked to membrane-bound G proteins, which are also linked to GDP and GTP. The key mechanism underlying these medicines' beneficial inotropic and chronotropic effects is their activation of GTP, which can work in either a stimulatory or an inhibitory manner [38]. Adrenergic receptors are regulated; the up directive occurs in reaction to a drop in adrenergic activity, and the down directive occurs in reaction to a rise in adrenergic activity. The concentration of receptors can change quickly, even within a few hours [38].

Phosphodiesterase Inhibitors

For immediate therapy to decrease cardiac output, milrinone is the only phosphodiesterase inhibitor currently available. cAMP metabolism is carried out by the enzyme phosphodiesterase III, which is inhibited by this substance. Milrinone is mild inotropic and vasodilatory effects causing it to lower the ventricle fill-up demands while enhancing cardiac output [39].

Nitric Oxide Donors: Nitroprusside and Nitroglycerin

Nitric oxide and cyanide anions are linked to sodium salt, which is then converted into nitroprusside. After interacting with red blood cells and some proteins, nitric oxide is released, inducing the production of cyclic guanosine monophosphate by guanyl cyclase and inhibiting the contraction of smooth muscle. On venous capacitance and arterial resistance vessels, nitroprusside has a dose-dependent vasodilatory effect [40]. The greatest danger of cyanide poisoning is when using nitroprusside in excess dose or administering it for an extended period [41].

Calcium Sensitizers

Nowadays, the most commonly used drug is levosimendan due to its various mechanisms of action, that is, by inhibiting phosphodiesterase III, increasing the calcium sensitivity of cardiac troponin C, and stimulating the opening of potassium adenosine triphosphate channel, which leads to its vasodilatory effect [42]. It is a pyridazine-dinitrile derivative. The typical intravenous levosimendan dosage used in a clinical trial for patients of HF is a loading dose of 6 to 12 microg/kg given over 10 minutes, followed by a continuous infusion of 0.05 to 0.2 microg/kg/minute. The hemodynamic response is typically noticed within 5 minutes of infusion of the loading dose. Levosimendan effects reach their peak at 10 to 30 minutes of infusion; due to active metabolite, the duration of action is roughly 75 to 78 hours to one week. There are various side effects, which include hypotension, arrhythmia, myocardial ischemia, hypoxemia, headache, dizziness, and nausea due to which levosimendan is used for short-term treatment of acute decompensated CHF [42].

ACTION

ACTION is the first pediatric ventricular assist device (VAD) that is widely used nowadays to improve the quality of life (QoL). There are still few treatments available for people with end-stage HF, despite the rising prevalence of acute and chronic HF in children. Before the development of VADs, the mortality rate was very high, especially in younger patients [43]. Now, results were much improved when VAD were used in patients of HF as compared to the pre-VAD era. The use of VADs increased from about 4% to 33% as a result of the outcomes improvement. The Institute of Medicine created ACTION in 2007 as a learning network to improve the clinical state of children with HF. More recently, ACTION was developed in 2017. Bleeding and infections are the two major adverse effects seen during the first month following the implantation of a VAD [43].

International Society of Heart and Lung transplantation 

The 2019 International Society for Heart and Lung Transplantation (ISHLT) study states that today, a VAD is used to bridge more than one-third of patients having transplants. The use of VADs as a transitional measure before transplant is a growing trend. The primary goal of VAD therapy is to stabilize the hemodynamics of a failing circulation that is not responding to conventional treatment. The VAD should be placed before the development of severe end-organ dysfunction to maximize therapeutic effects. The objective is to enhance the QoL, enhance tissue and organ perfusion, and enhance patient survival. The need for a consensus statement on the selection and treatment of children and adults with CHD receiving VAD implantation has been acknowledged by ISHLT [44].

ISHLT has stated guidelines for patient selection, which include the timing of the VAD, indication for VAD, intent of VAD, pre-implant planning, device selection, operative management, postoperative hemodynamic goals, and adverse events following VAD implantation. The various indications for VAD include failure in medical management, postcardiotomy failure to wean from cardiopulmonary bypass (CPB), and arrhythmias. The important steps in pre-implant planning include end-organ assessment, right heart assessment, support-type assessment, psychosocial assessment, and device fit. The devices used for short-term VAD in children are Rotaflow (Getinge, Harlingen, Germany), Pedimag (Abbott Laboratories, Chicago, IL, USA), Centrimag (Abbott Laboratories), Tandem Heart (LivaNova, Pittsburgh, PA, USA), Tandem Life ProtekDuo (LivaNova), and Impella RP (Abiomed, Danvers, MA, USA). For long-term VAD, Berlin Heart EXCOR (Berlin Heart, Berlin, Germany) and Heartmate 3 (Abbott Laboratories) system devices are used [44].

## Conclusions

This research contains all the latest information about drugs used in CHF and their mechanisms of action. A completely different spectrum of diseases, including CHD rather than cardiomyopathy, are responsible for pediatric HF. HF can be prevented from developing by early identification of risk factors and the initiation of proper therapy. Initially, an adaptive response occurring to ventricular dysfunction sets off a cascade of modifications that result in compensatory hypertrophy, which over time, if unchecked, leads to fibrosis and ventricular failure. Pediatric HF therapy includes surgical palliation and repair, as postoperative cardiac lesions that are left untreated might result in persistent HF. Although neurohumoral stimulation is similar in children to that in adults with HF, there are significant physiological variations between children and adults, notably regarding alterations in development that are most noticeable in newborns. A significant number of cardiovascular medications that have a solid database for lowering the hazards of unfavorable cardiac outcomes are selective beta 1 adrenoceptor inhibitors. Implementation of medical therapy in individuals with HF with reduced ejection fraction is difficult as these patients' physiological factors and comorbidities restrict the up-titration of lifesaving drugs. Lifestyle factors like poor diet, lack of physical exercise, obesity, and increased mental and emotional stress also contribute to developing HF. The availability, cost, and prescription policies of medications may also restrict patient access to them, with particular concerns arising in different areas over the globe.
